# Improved Diagnostic Imaging of Brain Tumors by Multimodal Microscopy and Deep Learning

**DOI:** 10.3390/cancers12071806

**Published:** 2020-07-06

**Authors:** Johanna Gesperger, Antonia Lichtenegger, Thomas Roetzer, Matthias Salas, Pablo Eugui, Danielle J. Harper, Conrad W. Merkle, Marco Augustin, Barbara Kiesel, Petra A. Mercea, Georg Widhalm, Bernhard Baumann, Adelheid Woehrer

**Affiliations:** 1 Center for Medical Physics and Biomedical Engineering, Medical University of Vienna, 1090 Vienna, Austria; johanna.gesperger@meduniwien.ac.at (J.G.); antonia.lichtenegger@meduniwien.ac.at (A.L.); matthias.salas@meduniwien.ac.at (M.S.); pablo.euguiarrizabalaga@meduniwien.ac.at (P.E.); danielle.harper@meduniwien.ac.at (D.J.H.); conrad.merkle@meduniwien.ac.at (C.W.M.); marco.augustin@meduniwien.ac.at (M.A.); 2 Division of Neuropathology and Neurochemistry, Department of Neurology, Medical University of Vienna, 1090 Vienna, Austria; thomas.roetzer@meduniwien.ac.at (T.R.); adelheid.woehrer@meduniwien.ac.at (A.W.); 3 Department of Neurosurgery, Medical University of Vienna, 1090 Vienna, Austria; barbara.kiesel@meduniwien.ac.at (B.K.); petra.mercea@meduniwien.ac.at (P.A.M.)

**Keywords:** optical coherence tomography, glioma, metastasis, attenuation

## Abstract

Fluorescence-guided surgery is a state-of-the-art approach for intraoperative imaging during neurosurgical removal of tumor tissue. While the visualization of high-grade gliomas is reliable, lower grade glioma often lack visible fluorescence signals. Here, we present a hybrid prototype combining visible light optical coherence microscopy (OCM) and high-resolution fluorescence imaging for assessment of brain tumor samples acquired by 5-aminolevulinic acid (5-ALA) fluorescence-guided surgery. OCM provides high-resolution information of the inherent tissue scattering and absorption properties of tissue. We here explore quantitative attenuation coefficients derived from volumetric OCM intensity data and quantitative high-resolution 5-ALA fluorescence as potential biomarkers for tissue malignancy including otherwise difficult-to-assess low-grade glioma. We validate our findings against the gold standard histology and use attenuation and fluorescence intensity measures to differentiate between tumor core, infiltrative zone and adjacent brain tissue. Using large field-of-view scans acquired by a near-infrared swept-source optical coherence tomography setup, we provide initial assessments of tumor heterogeneity. Finally, we use cross-sectional OCM images to train a convolutional neural network that discriminates tumor from non-tumor tissue with an accuracy of 97%. Collectively, the present hybrid approach offers potential to translate into an in vivo imaging setup for substantially improved intraoperative guidance of brain tumor surgeries.

## 1. Introduction

Although diffuse gliomas comprise less than 1% of all newly diagnosed cancers, they account for substantial patient morbidity and mortality. Glioblastoma (GB), the most malignant and lethal type of glioma with a median overall survival of only 15 months, comprises 70–75% of all gliomas [1,2,3]. In addition to this rare tumor entity, brain metastases (MET) are the most common type of central nervous system (CNS) tumors, estimated to occur in 9–10% of all cancer diagnoses with lung cancer, breast cancer and melanoma being the leading causes for brain MET [4]. In most cases, METs result in a devastating prognosis with an estimated overall survival of less than a year despite optimal therapeutic approaches [5].

Maximal safe resection is of utmost importance when operating on intracranial tumors. During brain tumor surgery, real-time imaging plays a crucial role in the optimal resection of CNS tumors [6,7]. Intraoperative optical microscopy provides enhanced resolution to guide through the procedure and support the neurosurgeon in the resection of brain tumors. Moreover, magnetic resonance imaging (MRI)-based neuro-navigation is routinely applied for image guidance during brain tumor surgeries. However, the discrimination between non-tumor and tumor tissue remains challenging for the neurosurgeon, as brain shift results in progressive inaccuracy of neuro-navigation during surgery [8,9]. One potential solution to this problem is visualization of tumor tissue with 5-ALA-induced protoporphyrin IX (PPIX) fluorescence (FL), first described in the neurosurgical field by Stummer et al. in 1998 [10], approved by the European Medicines Agency (EMEA) in 2007, and the U.S. Food and Drug Administration (FDA) ten years later [11]. 5-ALA is orally administered to the patient three to four hours prior to induction of anesthesia. The malignant brain tumor tissue synthesizes porphyrins such as PPIX, resulting in the emission of fluorescence at the site of malignant tumor tissue. Thereby, the intraoperative delineation of tumor tissue and especially tumor margins is facilitated [12,13,14]. Fluorescence-guided tumor visualization has been shown to provide high sensitivity and specificity for GB, however its capabilities for the intraoperative detection of lower-grade gliomas (LGG) and/or secondary brain tumors are limited [13,15,16].

The gold standard for the diagnosis of brain tumors is the integrated morphological and molecular evaluation of tumor tissue. Although being a highly specific and sensitive method, it is prone to sampling bias, and is time consuming and cost intense. Just recently, research groups have established first convolutional neural networks (CNN) and deep learning algorithms for the non-invasive classification of brain tumors based on MRI [17,18]. Likewise, another group recently reported deep CNNs applied to intraoperative stimulated Raman histology for brain tumor classification [19]. Despite these promising approaches, the extensive tumor heterogeneity that underlies many brain tumor types such as diffuse glioma remains a major diagnostic and therapeutic challenge [20,21,22]. Hence, a better understanding of tumor heterogeneity and higher throughput sampling will be key to improving diagnostic accuracy and refining personalized therapeutic options [23].

Optical coherence tomography (OCT) is a non-destructive and label-free imaging modality, which was first introduced in the early 1990s [24,25]. The technique is based on the inherent backscattering of light within different tissues and permits a three-dimensional, tissue-preserving reconstruction of morphology down to the cellular level [26]. OCT has become a standard diagnostic tool in ophthalmology and has recently evolved in other fields such as neuroimaging [27]. For instance, OCT has been used to investigate neurodegenerative diseases such as Alzheimer’s disease [28,29,30,31] and also to visualize brain tumors [32,33,34,35,36,37,38]. Most recently, Almasian et al. published a feasibility study on in vivo intraoperative OCT for glioma surgery based on a cohort of six patients [39]. Another group described an AI-assisted approach to detect human glioma infiltration in situ with outstanding sensitivity (near 100%) and specificity (around 85%) [40]. As the evaluation of OCT data is computationally intense, first CNNs have been established to differentiate between different intracranial tumors and non-tumor-containing brain tissue based on texture features of OCT images [41]. Yashin et al. demonstrated cross-polarization OCT for glioma imaging both ex vivo and in vivo and evaluated signal intensities, homogeneity of intensity, attenuation rates, and uniformity of attenuation [42]. However, OCT imaging based solely on the scattering of light often lacks tissue-specific contrast [27].

In this study, we aimed to combine OCT-based microscopy (OCM) with high resolution FL imaging for the evaluation of intracranial tumors acquired through 5-ALA fluorescence-guided surgery. Using a multimodal hybrid OCM-FL prototype [43], we evaluated FL intensities, OCM attenuation (ATT) coefficients and cell density maps across three different brain tumor entities, namely LGG (including WHO grade II and III gliomas), GB, and MET. For a deeper understanding of the intra-tumoral heterogeneity of our samples, we used a swept-source OCT setup at 1060 nm [44] for large field-of-view (FOV) scans. All findings were validated against the gold standard histology. Ultimately, we constructed a CNN to predict the absence or presence of tumor in the tissue samples based on cross-sectional OCM intensity data, as these may be available in real-time during surgery.

## 2. Results

### 2.1. Comparison of In Vivo 5-ALA Readout with Ex Vivo Fluorescence, OCM and Histology

**Figure 1:**
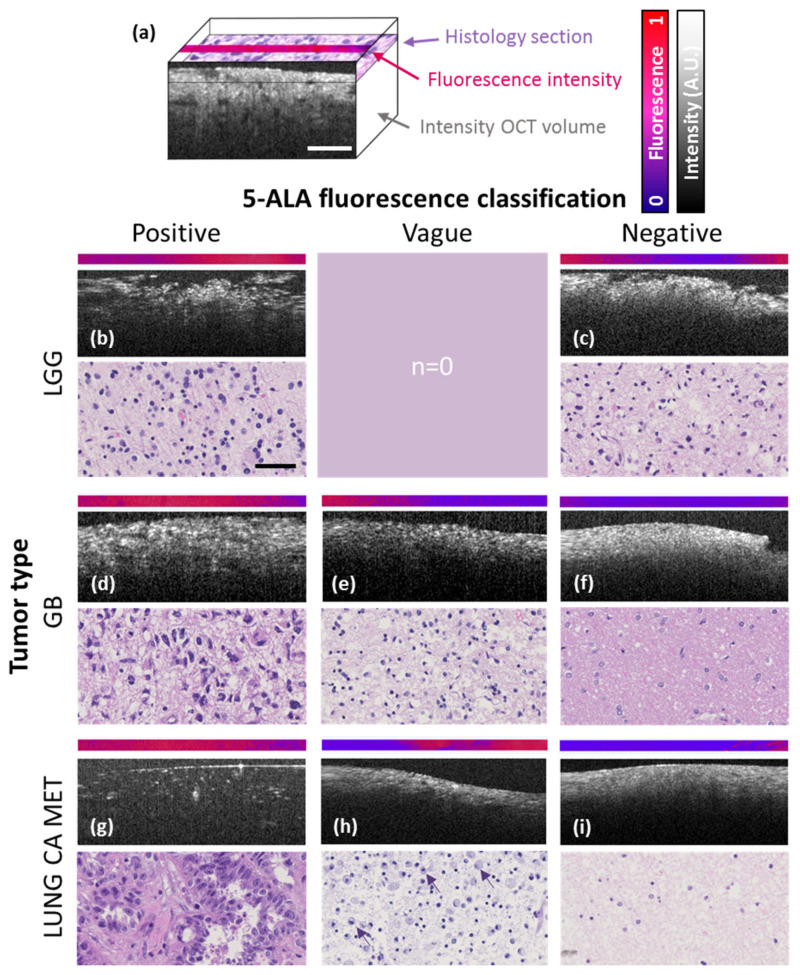
Cross-sections and digital pathology scans of representative tumor samples for each tumor entity evaluated in the study. Each panel features a 2.5-µm-thick line of the fluorescence micrograph and the corresponding OCM (optical coherence microscopy) B-scan image. Corresponding histological sections are shown below. (**a**) Schematic 3D representation provides orientation of feature extraction planes. (**b**,**c**) LGGs (lower-grade gliomas) with strong (**b**) and without (**c**) 5-ALA (5-aminolevulinic acid) fluorescence observed during fluorescence-guided surgery (FGS) respectively. (**d**–**f**) GB (glioblastoma) with strong (**d**), vague (**e**) and without (**f**) intraoperative 5-ALA fluorescence respectively. (**g**–**i**) Lung CA MET (lung cancer metastasis) with strong (**g**), vague (**h**) and without (**i**) 5-ALA fluorescence observed during FGS. Note, infiltration by macrophages in (**h**) indicated by purple arrows and necrosis in (**i**) throughout the tissue. Scale bars correspond to 50 µm.

In Figure 1a, a schematic representation provides orientation on the 3D arrangement of FL, OCM, and histologic imagery. The remaining Figure 1 highlights representative examples for all tumor entities evaluated in this study grouped according to the intraoperative 5-ALA fluorescence classification by the neurosurgeon. The pink-to-blue colored strips on top of each panel indicate the transverse high-resolution 5-ALA fluorescence profile over a range of 3 pixels as measured ex vivo at the same location of the OCM B-scan image below. At the bottom of each panel corresponding haematoxylin and eosin (H&E) stained histological sections are provided. In the left column, data from samples with strong intraoperative 5-ALA fluorescence from a lower-grade glioma (LGG), GB and lung cancer MET are shown. In Figure 1e,h, CNS tumors with vague fluorescence are displayed. In the right column, macroscopically 5-ALA negative tumor samples are shown. Even though the LGG sample was macroscopically classified 5-ALA negative by the neurosurgeon, the combined FL and OCM signature unravels distinct sub-visual fluorescence heterogeneities and tissue alterations. These observations suggest the presence of tumor tissue. This is further substantiated by histology that confirms the presence of malignant tumor cells (Figure 1c) rather than non-neoplastic brain tissue (Figure 1d). Moreover, high-resolution fluorescence values and cell density were positively correlated with cell proliferation as quantified by the MIB1 labeling index (LI), where a positive, statistically significant correlation was found (r_s_ = 0.05, *p*-value 0.040; r_s_ = 0.15, *p*-value 0.001).

### 2.2. Comparison of OCM Attenuation and Quantitative Fluorescence Data

**Figure 2:**
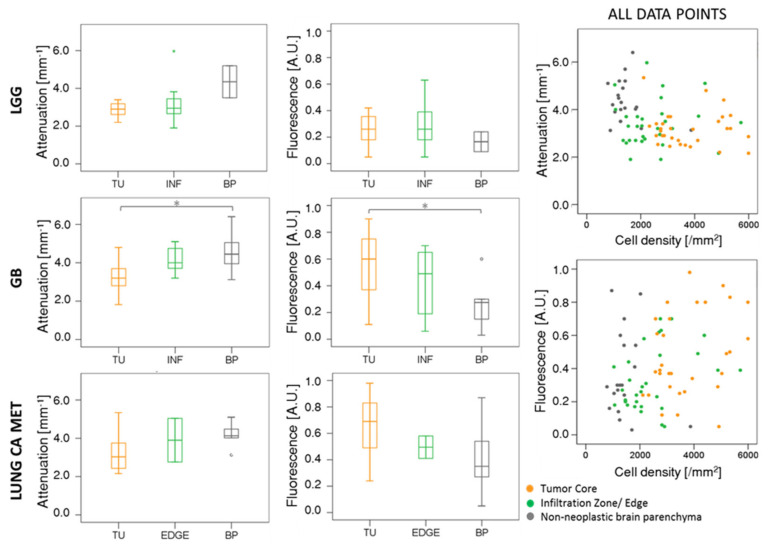
Comparison of median attenuation coefficients (mm^−1^ tissue depth) and normalized fluorescence intensities across different tumor entities. Scatter plots visualize every sample evaluated, where the axes represent attenuation coefficient and quantitative 5-ALA fluorescence with respect to cell density (cells/mm^2^). The color coding classifies the samples according to the tissue type assessed by an experienced neuropathologist based on histology. Fluorescence intensity values have been normalized between 0 and 1 (LGG–lower-grade glioma, GB–glioblastoma, LUNG CA MET–lung cancer metastasis, TU–tumor, INF–infiltration zone, BP–non-neoplastic brain parenchyma).

Next, attenuation and fluorescence data were compared for the different tumor entities (see Figure 2, Tables S2 and S3). For all three tumor entities, attenuation coefficients decreased while fluorescence signals increased with content of malignant tumor cells. For LGGs, the median attenuation coefficient in non-neoplastic brain parenchyma was 4.35 ± 1.20 mm^−1^, as compared to 2.95 ± 0.86 mm^−1^ and 2.90 ± 0.38 mm^−1^ in infiltration zone and tumor core, respectively. The relative 5-ALA fluorescence intensity was measured 0.16 ± 0.10 in non-neoplastic brain parenchyma, 0.25 ± 0.15 in infiltration zone and 0.26 ± 0.12 in the compact tumor. In the GB cohort, a significant difference between non-neoplastic brain parenchyma and tumor core of both attenuation coefficient (4.45 ± 0.99 mm^−1^ in non-neoplastic brain parenchyma, 3.20 ± 0.76 mm^−1^ in tumor, *p* = 0.04) and fluorescence intensity (0.28 ± 0.17 in non-neoplastic brain parenchyma, 0.60 ± 0.23 in tumor, *p* = 0.02) was found. For the infiltration/border zone, the attenuation coefficient was intermediate at 4.00 ± 0.71 mm^−1^ and the relative 5-ALA fluorescence was 0.49 ± 0.27. In the metastatic cohort, the same trends were observed for attenuation coefficients (4.13 ± 0.64 mm^−1^ in non-neoplastic brain parenchyma, 3.90 ± 1.61 mm^−1^ in the border zone, 3.03 ± 1.15 mm^−1^ in tumor) and fluorescence values (0.35 ± 0.28 in brain parenchyma, 0.50 ± 0.12 in border zone and 0.69 ± 0.27 in tumor). The scatter plots on the right-hand side of Figure 2 show attenuation coefficients and fluorescence intensities plotted over cell density independent of tumor entity. Each data point in the scatter plots corresponds to one data set.

### 2.3. Investigation of Intra-Tumor Heterogeneity Using Wide-Field OCM Imaging

**Figure 3:**
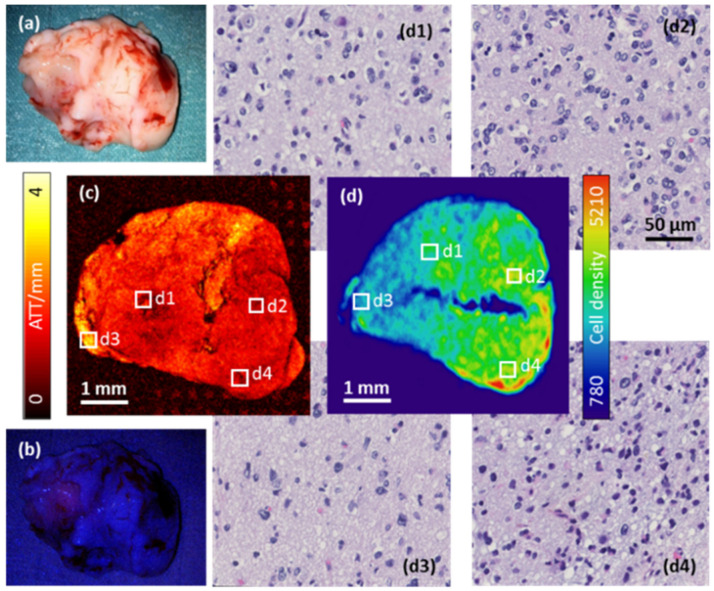
Large field-of-view (FOV) scan of a representative astrocytoma, WHO grade III case with a vaguely fluorescent 5-ALA hotspot. (**a**) Photograph and (**b**) fluorescence image of a sample of the compact tumor immediately after resection. (**c**) Attenuation en-face projection and (**d**) whole sample cell density map with (**d1**–**d4**) corresponding regions of interest upon H&E staining.

Large FOV images were acquired using the swept-source OCM setup in order to explore the intra-tumoral heterogeneity in a sub-cohort of six samples. Similar trends were observed in all tissue samples. A photograph (Figure 3a) and a fluorescence image (Figure 3b) of one sample derived from the core of an anaplastic astrocytoma, WHO grade III, are shown as a representative case. The intraoperative 5-ALA fluorescence classification was negative, suggesting the absence of highly malignant tissue. In Figure 3c, the attenuation en-face projection of the whole sample is displayed. Darker red tones represent lower attenuation coefficients, which seemed to be associated with more heterogeneous cell densities. Brighter colors ranging from orange to yellow encode a higher attenuation, which appears to correspond to less pathological tissue. Although the biopsies were all rather small (in the millimeter range), local variation of attenuation characteristics were observed. Four regions of interest of the size of 250 × 250 µm^2^ were chosen. The corresponding regions are highlighted in the cell density map in Figure 3d, and H&E stainings of the respective regions are shown in panels (d1) to (d4). Note that a slight tissue deformation was inevitable due to the histopathological workup including dehydration, embedding, and cutting processes. The mean cell density of the entire biopsy was 4477 cells/µm^2^, ranging from 3880 cells/µm^2^ in (d3) to 8267 cells/µm^2^ in (d4). Attenuation and cell density did not correlate with regional cell proliferation (as quantified by the Mindbomb homolog 1 (MIB1) score), Isocitrate Dehydrogenase 1 (IDH1), p53, or Epidermal Growth Factor Receptor (EGFR) expression. 

### 2.4. CNN for Tumor Classification in OCM Intensity B-Scan Data

**Figure 4:**
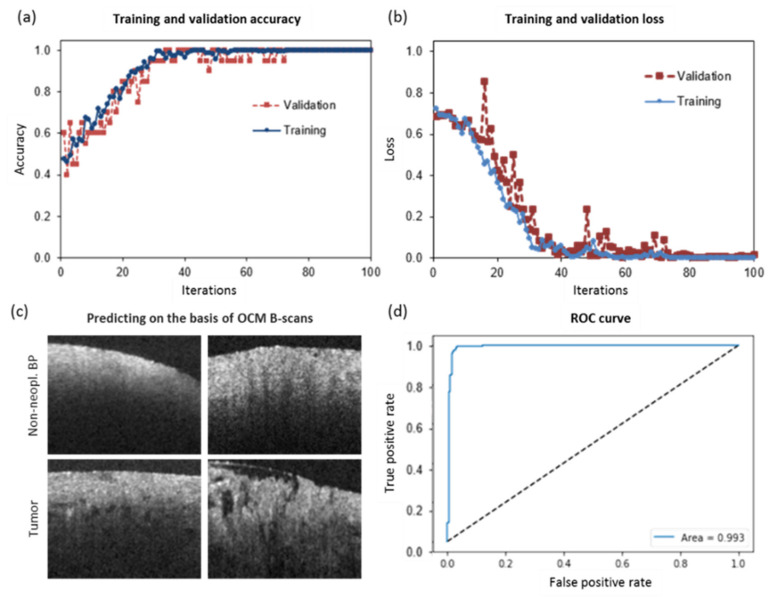
CNN (convolutional neural network)-based prediction of tumor versus non-neoplastic brain parenchyma. (**a**) Accuracy of training and validation processes over 100 iterations. (**b**) Loss during training and validation processes over 100 iterations. (**c**) Examples of OCM cross-sections of non-neoplastic brain parenchyma (Non-neopl. BP) versus tumor core. (**d**) ROC curve and area under the curve (AUC) of the CNN.

**Figure 5:**
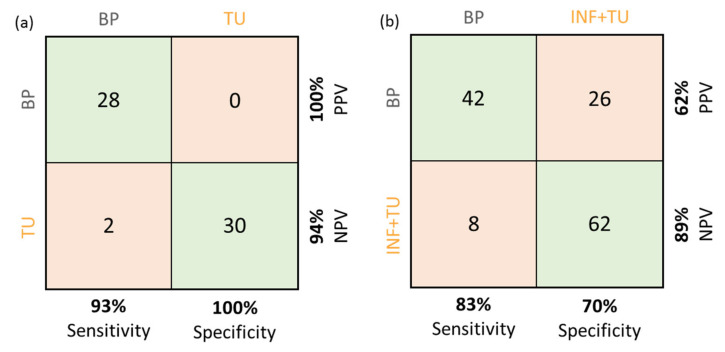
Contingency tables for both CNNs trained in this study. (**a**) Contingency table with positive predictive values (PPV) and negative predictive values (NPV) for the network trained on tumor (TU) and non-neoplastic brain parenchyma (BP) tissue B-scans. (**b**) Contingency table with PPV and NPV for the network trained on both infiltration zone and tumor (INF + TU) as compared to non-neoplastic tissue B-scans.

A CNN was trained to differentiate tumor from non-tumor affected brain tissue based on cross-sectional OCM intensity images, independent of fluorescence data. The model performance in terms of accuracy and loss (using categorical cross-entropy) is shown in Figure 4a,b, respectively. As a first attempt, the model was trained on tumor vs. non-neoplastic brain parenchyma (*n* = 204). When testing the model with an independent test set of 60 B-scans an accuracy of 97% was achieved. The sensitivity was found to be 93% and the specificity 100%. Representative OCM B-scans of non-neoplastic brain parenchyma and tumor tissue used for network training are shown in Figure 4c. Of note, differences in cell density, blood vessels, or axonal structures impact OCT signals. Figure 4d shows the ROC curve and the corresponding area under the curve (AUC = 0.993) of the CNN. As a next step, B-scans of samples resected from infiltration zones/tumor edge were included in the training data set (*n* = 257). The cross-sections with tumor infiltrative tissue were pooled with the tumor set. When testing the network using an independent test set of 138 unknown data sets, the accuracy dropped to 83% and the sensitivity and specificity were calculated to be 84% and 74%, respectively (see contingency tables in Figure 5). For the nonsense test set of 20 non-cerebral images, results showed a random group assignment with an accuracy of 50% as expected.

## 3. Discussion

In the present study, we investigated the potential of a combined multimodal fluorescence and optical coherence microscopy (OCM) setup for the characterization of brain tumor samples derived from 5-ALA fluorescence-guided surgery (FGS). Brain tumors carry a devastating prognosis and almost all of them either recur at some point or immediately progress to more malignant grades [45]. As more individualized therapeutic approaches are being increasingly incorporated, maximal safe resection is a major determinant of overall survival [46,47,48,49,50]. Hence, intraoperative guidance to support the neurosurgeon in performing complete resections has become increasingly relevant over the last decades. MRI-based neuro-navigation was established in the late 1990s and has since become a standard diagnostic tool [51]. Still, upon opening of the skull, intracranial pressure changes and resection of tumor tissue results in brain shift, rendering the images for intraoperative image guidance inaccurate [7,8,9], which is a limitation that can be avoided by intraoperative MRI. However, intraoperative MRI is costly and thus not widely available. 5-ALA-induced emission of PPIX fluorescence has been shown to be highly sensitive for high-grade gliomas and particularly GB [15,50,52,53]. However, FGS lacks sensitivity for LGG that mostly do not display visible fluorescence [54,55,56,57]. Further, unspecific fluorescence of erythrocytes and blood vessels might mimic true fluorescence signals, whereas necrotic tissue in a neoplastic cortex does not emit any fluorescence due to the terminated cell metabolism [58]. Hence, there is room to further develop or adapt novel technologies for intraoperative use such as OCM, which constitutes one particularly promising technique.

In our approach, we combined OCM with fluorescence imaging to simultaneously analyze OCM-based attenuation coefficients and quantitative high-resolution fluorescence using ex vivo tumor samples. For GB and MET, 5-ALA fluorescence, as assessed in vivo by the neurosurgeon as well as ex vivo by our combined system, was a rather specific predictor of tissue malignancy, well in line with the literature [9,11,55,59]. Of note, intra-operatively assessed macroscopic 5-ALA fluorescence was strongly correlated with the high-resolution fluorescence quantified by our prototype. Importantly however, our prototype showed enhanced sensitivity to detect even low-level sub-visual fluorescence, indicating that it might be especially useful for tumors with limited 5-ALA uptake or metabolism such as LGGs. LGGs typically show either no or only focally fluorescing areas, the latter being clinically important as they usually correspond to focal areas of malignancy [54,55,56,57]. Indeed, in our series, only 4 out of 32 regional LGG samples derived from tumor core or infiltration zones showed macroscopically visible 5-ALA fluorescence, a fraction that was substantially improved by our high-resolution fluorescence approach (12/32 samples). Five out of those 12 samples corresponded to anaplastic foci, thus rendering the patients candidates for more aggressive adjuvant therapy [60,61,62,63]. In contrast, the attenuation coefficient primarily depends on structural tissue properties or cellular characteristics, which makes it an especially attractive candidate to complement fluorescence. As a next step, it would be of interest to evaluate the ability of our prototype to classify those tumors according to their respective grades of malignancy in a larger study cohort.

Across our series of evaluated brain tumor types, we found that higher fluorescence values correlated with decreased attenuation values. In particular, attenuation coefficients decreased with increasing tissue heterogeneity and irregularities such as increased cell density. Of note, similar results for attenuation values have recently been observed by different research groups in the field [32,36,42]. Even though they used systems operating in the near infrared region, the overall trends were similar: attenuation coefficients decreased with increasing cell density, mostly corresponding to tumor malignancy. In our case, this was also true for LGG samples that did not display any visible 5-ALA fluorescence intraoperatively but still had histologically confirmed tumor infiltration, thus independently reproducing the results of our high-resolution fluorescence experiment.

In order to further tackle different microenvironmental compartments and intra-tumor heterogeneity in diffuse gliomas, we aimed for large FOV scans for a subgroup of our samples. In a previous paper we introduced a mosaicking-method using an automated x-y-z stage for large FOV scans at microscopic resolution [30]. However, this method is rather slow with an acquisition time of up to 8.3 seconds per FOV sized 200 × 200 µm^2^. In contrast, with our swept-source OCM setup we achieved a field of view of 8 × 8 mm^2^ in a single acquisition in only 5 seconds. Further, it offered an extended imaging depth range and an improved roll-off, which did not require manual refocusing for every tile. Even though the samples we analyzed represented only small parts of the overall tumor, attenuation coefficients and cell densities strongly varied within a single sample. This indicates that even within small samples, we face considerable tumor heterogeneity, making not only resection but also diagnostic assessments challenging.

As the calculation of attenuation coefficients requires computationally intense image processing, our next step was to evaluate the predictive potential of pre-stage cross-sectional reflectivity images as these can be obtained in real-time. To this end, we developed a convolutional neural network to differentiate between non-neoplastic brain parenchyma and tumor tissue based on the inherent backscattering of light within the sample. We did not differentiate between the individual tumor entities in our study in order to increase sample size and to evaluate the overall potential of the network to detect tumors independent of type. Indeed, we were successful in the prediction of the presence of tumor with high accuracy of 97%, which dropped to fair 83% when adding samples from the border/infiltration zone. This is to be expected considering the fact that the infiltration zone is a mixture of normal and tumor tissue. Still, considering the limited training sample size of 204 images used for this network, accuracies of 97% and 83% are an encouraging preliminary result. In future, the predictive accuracy might be further increased through the addition of high-resolution fluorescence images to the input data. Moreover, it will be important to evaluate its diagnostic performance under real-time frozen section conditions. The implementation of the proposed fluorescence imaging and optical coherence microscopy (FI-OCM) setup into the surgical theater might constitute a promising tool to intra-operatively discriminate different tissue types and optimize the extent of resection.

## 4. Materials and Methods

### 4.1. Tumor Samples

**Figure 6:**
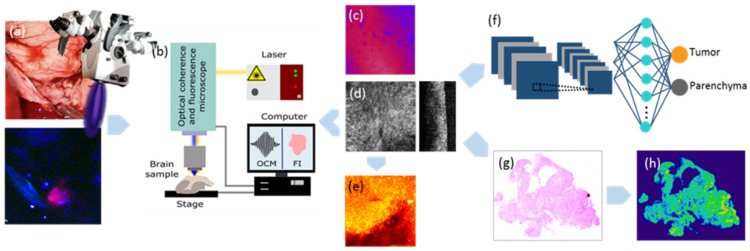
Workflow for the processing of CNS tumor biopsies. The samples were resected during 5-ALA FGS [13] (**a**) and immediately transferred to the lab, where FI-OCM imaging was performed (**b**). FI data were acquired before bleaching occurred (**c**), followed by OCM imaging (**d**). Based on OCM intensity data, attenuation maps were computed (**e**). Data post-processing was performed and all OCM data sets were evaluated using a convolutional neural classifier model (**f**). Subsequently, the samples were fixed in 4% formalin and processed for histopathological workup. H&E staining (**g**) and cell density maps (**h**) served as ground truth.

A total of 78 tissue samples retrieved from 42 patients from the vital tumor core (TU) and infiltration zone/edge (INF) with a diameter ranging from three to eight millimeters were safely collected during 5-ALA FGS of brain tumors and the performing neurosurgeon subjectively classified each sample according to the visible fluorescence level (strong, vague, or no fluorescence). We also collected samples from non-neoplastic brain parenchyma (BP) adjacent to the brain tumor, particularly en route to deep-seated tumors. Subsequently, all samples were grouped into three different categories based on the neuropathological diagnosis following the integrated morphological and molecular assessment according to the 2016 WHO classification [64]: LGG (*n* = 34), GB (*n* = 30), and lung cancer (CA) MET (*n* = 14; see Table 1 and Table S1 for demographic patient data). LGGs included eleven oligodendroglioma specimens (six of WHO grade II and five of WHO grade III) as well as 23 astrocytoma specimens (14 of WHO grade II and nine of WHO grade III). Features of interest included quantitative 5-ALA FL (normalized between 0 and 1), attenuation (ATT) coefficients (mm^−1^) and cell density (cells/mm^2^). Fresh intraoperative samples were safely collected and imaged within one hour after resection using the custom-built hybrid OCM and FL imaging setup (see the workflow scheme in Figure 6). A sub-population of six samples was further imaged with the swept-source OCM system described below to acquire large FOV scans. The tissue samples were acquired from FGS performed at the Department of Neurosurgery of the Medical University of Vienna, Austria (approved by local ethics committee of the Medical University of Vienna, Austria; approval number EK 419/2008—Amendment 04/2018). The histopathological analyses were conducted at the Division of Neuropathology and Neurochemistry, Department of Neurology of the Medical University of Vienna, Austria.

### 4.2. Optical Coherence Microscopy and Fluorescence Imaging

For the investigation of our study cohort, a custom-built multimodal hybrid OCM and FL imaging system was utilized. A detailed description of the system can be found elsewhere [43]. In brief, the same FOV was sequentially imaged with both modalities. A visible supercontinuum laser (NKT Photonics, SuperK EXTREME EXU-6, Birkerod, Denmark) was used as light source for both OCM and FI systems. Switching from OCM to FL imaging only required flipping two mirrors in the setup. Fluorescence images were acquired prior to OCM volumes in order to prevent artifacts due to the bleaching process. The FL subsystem was based on a confocal laser scanning microscope configuration employing a filter cube equipped with an excitation filter (Thorlabs, Luebeck, Germany; MF 434-17), a dichroic mirror (Thorlabs, MD 434), an emission filter (Thorlabs, MF 630-69) and a photomultiplier tube (Thorlabs, PMMT02). The OCM subsystem used a broad light spectrum in the visible range (425–685 nm) providing an ultrahigh axial resolution of 0.88 µm in brain tissue. The measured lateral resolution was 1.8 µm using a 20× objective lens (Olympus, Tokyo, Japan; UPLFLN-20XP). The power measured at the sample was 0.8 mW for OCM and 0.2 mW for FL imaging.

### 4.3. Data Acquisition and Post-Processing with the Multimodal OCM Setup

Fluorescence images consisted of 500 (x) × 500 (y) pixels and the processed OCM data sets comprised 500 (x) × 500 (y) × 4096 (z) pixels, each covering the same 200 × 200 µm2 lateral FOV. OCM volumes and FL intensities were acquired in 8.3 seconds using custom software (LabVIEW 2015, Version 15.0, 64-bit, National Instruments, Austin, TX, USA). Data were processed as previously described by Lichtenegger et al. [30]. After image acquisition, surface flattening was performed and attenuation maps were computed from OCM reflectivity data [65,66]. Sub-volumes consisting of 100 B-scans were chosen in a manually defined region of interest and average attenuation coefficients were calculated. Respective fluorescence intensities on the tissue surface were quantified and normalized linearly between zero and one, with the upper and lower values defined as the minimum and maximum, respectively, of all data sets. For visualization purposes, Fiji [67] was used to generate averaged en-face projections and composition images.

### 4.4. Large Field-Of-View Scans

For a deeper insight into the intra-tumoral heterogeneity of our samples, multisector imaging was performed for six representative samples. For this purpose, a modified configuration of a swept-source OCT ophthalmoscope previously introduced by Salas et al. [44] was used. The light source (Insight Photonic Solutions, Inc., Lafayette, CO, USA) operated at 100 kHz and provided a bandwidth of 73 nm centered at 1060 nm. The measured axial resolution was 5.9 µm in brain tissue. With a FOV of 8 × 8 mm^2^, a transverse resolution of 38 µm was achieved.

### 4.5. Histopathological Workup

After OCM and FL imaging, tissue samples were immediately processed for histopathological workup. The tissue was fixed in 4% formalin for 24 to 48 hours depending on the size of the tumor sample, followed by dehydration steps and paraffin embedding. Next, 2.5-µm-thick sections were cut and hematoxylin & eosin (H&E; hematoxylin by Merck, Darmstadt, Germany; LOT HX90507449; eosin by Merck, CAS 17372-87-1) as well as Ki-67 antibody MIB1 (dilution 1:100, by Dako, Jena, Germany; LOT 20062304) staining was performed. Slides were digitized using a Hamamatsu (Hamamatsu City, Japan) NanoZoomer 2.0 HT slide scanner. Histological sections served as gold standard for correlations to OCM and FL imaging and were further used for cell density calculations.

### 4.6. Cell Density Maps and Statistical Correlation

Using a custom-made algorithm implemented in MATLAB (release R2014b, MathWorks, Natick, MA, USA), cell density calculations were performed and heat maps were created for each sample [68]. Cells were segmented by first performing color deconvolution on H&E stained slides to obtain an 8-bit grayscale image of the hematoxylin color channel [67,69,70]. Afterwards, automated thresholding was performed based on Phansalkar’s method [71]. Results were correlated to ATT coefficients and FL values using one-way ANOVA and Bonferroni corrections. In a next step, Spearman’s rank-order correlations were performed for MIB1 LI proliferation scores of all samples. Results were considered significant at the 0.05 level. All statistical evaluations were performed either using SPSS (version 24, IBM) or MATLAB (R2019b, The MathWorks).

### 4.7. Convolutional Neural Network

**Figure 7:**
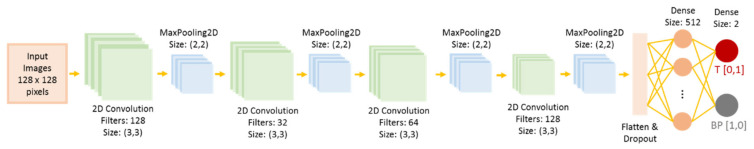
The architecture of the convolutional neuronal network. MaxPooling2D—2D extraction of most active (max) value from each cluster of neurons; Dense—dense blocks; T—tumor; BP—non-neoplastic brain parenchyma.

A convolutional neural network (CNN) classifier model was coded in Python (version 3.6) using Keras libraries (version 2.2.4) and TensorFlow (version 2.0) as a backend. A sequential model was built using 2D-convolution, max-pooling, dropout and dense blocks. An optimizer was used for compiling. An overview of the CNN architecture is given in Figure 7. First, a total of 204 reflectivity B-scans of non-neoplastic brain parenchyma (*n* = 102 retrieved from 18 patients using five to six B-scans per patient) and compact tumor tissue (*n* = 102 retrieved from 18 patients using five to six B-scans per patient) acquired with our visible light OCM setup were used as data set for training the model. The tumor samples included all tumor entities incorporated in this study (LGG, GB, MET). Each OCM B-scan was scaled to 128 × 128 pixels in size. All grayscale values in the OCM B-scans were normalized between 0 and 1. In order to split the images into training and validation sets, 10-fold cross validation was performed. Each time before training, the data set was randomly shuffled on a sample-basis in a way that the same sample could not be used for both training and validation. An Adam optimizer was applied in addition to a dropout of 0.5 and 100 epochs were trained. After training, prediction analyses were performed for an independent test data set of 60 data B-scans (*n* = 30 for compact tumor core and *n* = 30 for non-neoplastic tissue adjacent to the tumor, respectively). The prediction and training sets were taken from independent patient data. A label matrix (0—non-neoplastic brain parenchyma, 1—tumor core) was predefined based on histological classification. Additionally, 20 randomly selected OCM B-scan data sets of ex vivo tissue other than human CNS tissue (cornea, mouse brain and zebrafish larvae tissue) were tested with the neuronal network to test its specificity. Second, all data sets including infiltrative regions (that are a mixture of brain parenchyma and low-cellular/scant tumor) were investigated using the same network architecture. The network was trained again with 10-fold cross validation using 118 and 157 data sets from non-neoplastic brain parenchyma (BP) and tumor tissue (TU), respectively. The infiltration zone data were added to the tumor data sets for training to test the extent of sensitivity of the network in terms of tumor margin detection. The network was then tested on 138 new data sets including B-scans from the tumor core, infiltration zone, and tumor-associated BP.

### 4.8. Data and Code Availability Statement

All data and code used in this study will made available upon direct request. Clinical data will be provided fully anonymized. None of the data may be used for commercial purposes. This policy complies with the requirements of the funding sources.

## 5. Conclusions

We present a multimodal hybrid FL-OCM setup and assess regional samples across a range of different brain tumor types derived from 5-ALA FGS. The combined evaluation of high-resolution fluorescence intensities and OCM-derived attenuation coefficients proves powerful and synergistic for evaluating challenging samples that do not display visible 5-ALA fluorescence such as lower grade glioma. Finally, we use standard OCM image data to differentiate between tumor and non-neoplastic brain tissue using a deep learning-based approach. Altogether, our hybrid imaging prototype and image analysis algorithm are promising tools for improved intraoperative guidance to achieve the surgical goal of maximal safe resections of brain tumors.

## Figures and Tables

**Table 1:** Patient and biopsy cohort with respect to tumor entity (LGG, GB, MET) and histology-derived tissue type (TU, INF/EDGE, BP).

Tumor Entity	Compact Tumor Tissue	Infiltration Zone/Edge	Non-Neoplastic Tissue Adjacent to Tumor
Lower-grade glioma	9 patients, 11 biopsies	13 patients, 21 biopsies	2 patients, 2 biopsies
Glioblastoma	14 patients, 15 biopsies	4 patients, 7 biopsies	7 patients, 8 biopsies
Lung cancer metastasis	5 patients, 6 biopsies	2 patients, 2 biopsies	6 patients, 6 biopsies

## Supplementary Materials

The following are available online at https://www.mdpi.com/2072-6694/12/7/1806/s1, Table S1. Clinical patient data with patient ID, age in years (yrs), gender (f–female, m–male), location and diagnosis according to the 2016 WHO Classification of Tumors of the Central Nervous System, Table S2. Image parameters according to histological glioma subtype, Table S3. Image parameters according to IDH mutation status.
