# Supplementary material for: Improved Diagnostic Imaging of Brain Tumors by Multimodal Microscopy and Deep Learning
Source: Cancers (Basel). 2020 Jul 6;12(7):1806. doi: 10.3390/cancers12071806 (PMC7408054; doi:10.3390/cancers12071806)
Supplement: Supplementary file 1 [file cancers-12-01806-s001.pdf]

cancers

MDPI

Supplementary Materials

Table S1. Clinical patient data with patient ID, age in years (yrs), gender (f – female, m – male), location and diagnosis according to the 2016 WHO Classification of Tumors of the Central Nervous System.

Patient ID	Age	Gender	Location	Diagnosis
1	73 yrs	m	Frontal right	Secondary glioblastoma, IDH mutant (WHO IV)
2	50 yrs	m	Intraaxial temporal	Glioblastoma, IDH wildtype (WHO IV)
3	56 yrs	f	Insular	Anaplastic oligodendroglioma, IDH mutant and 1p/19q co-deleted (WHO III)
4	73 yrs	f	Intraaxial occipital	Glioblastoma, IDH wildtype (WHO IV)
5	55 yrs	f	Frontal	Lung cancer metastasis
6	60 yrs	f	Intraaxial occipital	Lung cancer metastasis
7	67 yrs	f	Cerebellar	Lung cancer metastasis
8	58 yrs	m	Intraaxial frontal right	Lung cancer metastasis
9	48 yrs	f	Parietal left	Glioblastoma, IDH wildtype (WHO IV)
10	38 yrs	m	Temporal left	Diffuse astrocytoma, IDH mutant (WHO II)
11	41 yrs	f	Intraaxial insular right	Oligodendroglioma, IDH mutant and 1p/19q co-deleted (WHO II)
12	40 yrs	f	Intraaxial parietal left	Diffuse astrocytoma, IDH mutant (WHO II)
13	46 yrs	f	Frontal left	Anaplastic astrocytoma, IDH mutant (WHO III)
14	42 yrs	m	Frontotemporal right	Glioblastoma, IDH wildtype (WHO IV)
15	53 yrs	m	Parietal left	Glioblastoma, IDH wildtype (WHO IV)
16	55 yrs	f	Gyrus cinguli	Diffuse astrocytoma, IDH mutant (WHO II)
17	39 yrs	f	Frontal right	Glioblastoma, IDH wildtype (WHO IV)
18	78 yrs	f	Frontal left	Diffuse astrocytoma, IDH mutant (WHO II)
19	75 yrs	f	Intraaxial temporal	Secondary glioblastoma, IDH mutant (WHO IV)
20	67 yrs	m	Intraaxial temporal left	Glioblastoma, IDH wildtype (WHO IV)
21	38 yrs	m	Intraaxial parieto-occipital left	Glioblastoma, IDH wildtype (WHO IV)
22	30 yrs	m	Prepostcentral	Anaplastic astrocytoma, IDH mutant (WHO III)
23	88 yrs	m	Frontal left	Glioblastoma, IDH wildtype (WHO IV)
24	72 yrs	m	Temporal right	Glioblastoma, IDH wildtype (WHO IV)

25	60 yrs	f	Intraaxial	Control
26	42 yrs	m	Intraaxial temporal	Anaplastic oligodendroglioma, IDH mutant and 1p/19q co-deleted (WHO III)
27	68 yrs	m	Intraaxial temporal right	Gliosarcoma, IDH wildtype (WHO IV)
28	52 yrs	m	Parietal	Oligodendroglioma, IDH mutant and 1p/19q co-deleted (WHO II)
29	65 yrs	f	Intraaxial temporal	Anaplastic astrocytoma, IDH wildtype (WHO III)
30	43 yrs	f	Intraaxial frontal right	Anaplastic glioma, IDH wildtype, NOS (WHO II)
31	50 yrs	f	Intraaxial frontal left	Lung cancer metastasis
32	62 yrs	m	Hemispherical right	Glioblastoma, IDH wildtype (WHO IV)
33	62 yrs	m	Frontal	Anaplastic astrocytoma, IDH mutant (WHO III)
34	52 yrs	m	Intraaxial temporal	Anaplastic oligodendroglioma, IDH mutant and 1p/19q co-deleted (WHO III)
35	53 yrs	m	Intraaxial	Oligodendroglioma, IDH mutant and 1p/19q co-deleted (WHO II)
36	80 yrs	m	Temporal	Secondary glioblastoma, IDH mutant (WHO IV)
37	67 yrs	m	Intraaxial frontal	Glioblastoma, IDH wildtype (WHO IV)
38	34 yrs	m	Intraaxial temporal right	Diffuse astrocytoma, IDH mutant (WHO II)
39	75 yrs	m	Intraaxial occipital	Glioblastoma, IDH wildtype (WHO IV)
40	55 yrs	m	Cerebellar	Lung cancer metastasis
41	31 yrs	f	N.A.	Glioblastoma, IDH wildtype (WHO IV)
42	75 yrs	f	Intraaxial parietal	Lung cancer metastasis

Table S2. Image parameters according to histological glioma subtype.

Tissue Type	Parameter	Oligodendroglioma (WHO II and III, *n* = 11)	Astrocytoma (WHO II and III, *n* = 23)
Brain parenchyma (*n* = 1 for ODG and AC)	Median attenuation	5.20 mm^{-1}	3.50 mm^{-1}
	Median fluorescence	0.24	0.09
	Median cell density	1422/mm^2	1251/mm^2
Infiltration zone	Median attenuation	3.10 ± 0.47 mm^{-1}	3.00 ± 0.99 mm^{-1}
	Median fluorescence	0.32 ± 0.17	0.28 ± 0.15
	Median cell density	2482 ± 1670/mm^2	2362 ± 845/mm^2
Tumor core	Median attenuation	3.2 ± 0.21 mm^{-1}	2.70 ± 0.39 mm^{-1}
	Median fluorescence	0.31 ± 0.14	0.23 ± 0.11
	Median cell density	2868 ± 166/mm^2	3550 ± 788/mm^2

Table S3. Image parameters according to IDH mutation status.

Tissue Type	Parameter	IDH Wildtype (*n* = 5)	IDH Mutant (*n* = 29)
Brain parenchyma	Median attenuation	n = 0	4.35 ± 1.20 mm^{-1}
	Median fluorescence	n = 0	0.17 ± 0.11
	Median cell density	n = 0	1336 ± 121/mm^2
Infiltration zone	Median attenuation	2.17 ± 0.46 mm^{-1}	3.24 ± 0.82 mm^{-1}
	Median fluorescence	0.42 ± 0.08	0.27 ± 0.15
	Median cell density	1982 ± 660/mm^2	2466 ± 1552/mm^2
Tumor core	Median attenuation	2.31 ± 0.21 mm^{-1}	3.01 ± 0.30 mm^{-1}
	Median fluorescence	0.16 ± 0.15	0.28 ± 0.11
	Median cell density	4287 ± 897/mm^2	3083 ± 478/mm^2
